# Improving the Detection Ability of Inductive Micro-Sensor for Non-Ferromagnetic Wear Debris

**DOI:** 10.3390/mi11121108

**Published:** 2020-12-15

**Authors:** Man Wang, Haotian Shi, Hongpeng Zhang, Dian Huo, Yucai Xie, Jun Su

**Affiliations:** Marine Engineering College, Dalian Maritime University, Dalian 116026, China; Wangman@dlmu.edu.cn (M.W.); dmu6hao@dlmu.edu.cn (H.S.); hd116026@163.com (D.H.); xyc86418332@dlmu.edu.cn (Y.X.); sujun738192099@163.com (J.S.)

**Keywords:** non-ferromagnetic debris, detection ability, inductance parameter, resistance parameter, excitation frequency

## Abstract

The inductive debris sensor has been studied because of its wide application prospects in mechanical health monitoring. In order to ensure a high-precision detection performance, a comprehensive method to improve the detection sensitivity and detection ability of the inductive sensor for non-ferromagnetic metal debris is proposed. Based on the characteristics of the eddy current inside the metal, the change of the coil impedance caused by the metal debris is increased by enhancing the magnetic field strength and selecting the optimal excitation frequency. The impedance detection method involving inductance and resistance parameters is used to improve the detection limit of non-ferromagnetic metal debris. The experimental results verify that the magnetic field in the detection region can be enhanced by adding a silicon steel strip (paramagnetic material) in the central hole of the coil, thereby greatly improving the detection sensitivity of the inductive sensor, and the concentrated distribution of the magnetic field avoids the double-peak signals generated by a single particle. The characteristics of the signal amplitude of non-ferromagnetic debris with excitation frequency are studied. Higher inductance, resistance amplitudes, and signal-to-noise ratio (SNR) can be obtained by using a high-frequency alternating current. Compared with inductance parameter detection, resistance parameter detection can detect smaller non-ferromagnetic debris. Combining the detection results of the inductance and resistance parameters can effectively improve the sensor’s ability to detect non-ferromagnetic debris.

## 1. Introduction

Oil wear debris monitoring is an effective method of mechanical condition monitoring. According to the characteristics of wear debris in oil, the wear degree, type, and position of equipment parts can be analyzed and judged. This technology has been used in the condition monitoring of rotating and reciprocating equipment such as aircraft engine bearings [1], marine hydraulic equipment [2], wind turbine gearboxes [3], etc., to achieve system fault diagnosis and life prediction. Based on the real-time oil monitoring results, the operators can repair and replace the oil to ensure the safe operation of the equipment and prolong remaining useful life. Over the past decades, oil condition monitoring technology with different detection principles is studied, mainly including ferrographic analysis [4,5], spectroscopy analysis [6,7], acoustic detection [8,9], imaging method [10,11], capacitance detection [12,13], and inductance detection [14,15]. Among the above methods, inductance detection is a non-destructive method based on the principle of electromagnetic induction. It can not only distinguish the properties of wear debris, but also, the results are not affected by the light transmittance, pH, temperature, and other impurities (air bubbles, water, and so on) in oil [16,17,18]. Therefore, most oil condition monitoring devices use inductance sensors as the core sensing element.

Recently, researchers have designed inductance sensors with various structures based on the 3D-solenoid coil and planar coil. The detection accuracy of the sensors is improved by optimizing the measurement circuit, enhancing the magnetic field of the detection region, and using the signal processing. Du et al. [19] attempted a parallel LC resonance circuit with unique resonant frequency to improve the SNR and sensitivity of the inductive sensor. Ren et al. [20] designed an effective unbalance compensation circuit to prevent the asymmetry of the excitation coils from limiting the sensor’s sensitivity, so as to enhance the useful signal and its stability for better detection results. Zhang et al. [21] presented a debris sensor with the structure of two planar coils in parallel, and a detection region with a high-gradient magnetic field is established by the two excitation silicon steel strips and a built-in silicon steel strip; this sensor can detect and distinguish 25 μm iron particles and 85 μm copper particles. Feng et al. [22] introduced an inductive sensor consisting of a cylindrical core, two L-shaped magnetic poles, an excitation coil, and an induction coil, and experiments indicated that the sensor can identify 25 μm ferromagnetic particles by the direct current driving. Hong et al. [23] proposed a hybrid method that combined a band pass filter and correlation algorithm to detect smaller debris with the same SNR. Based on a modified lock-in amplifier and empirical mode decomposition and reverse reconstruction, Zheng et al. [24] extracted the particle signals from the raw signal with an extremely low SNR, which significantly improves the sensor’s sensitivity. Of course, the inductive debris sensor can also be integrated with other debris detection technologies to enhance the detection ability [25,26]. 

Inductance detection can distinguish between ferromagnetic debris and non-ferromagnetic debris through the magnetization effect and eddy current effect generated by the metal debris in the magnetic field. However, the inductive sensor’s ability to detect non-ferromagnetic debris is relatively weak, which affects the sensor’s comprehensive detection performance. For the same volume of particles, the weakening of a magnetic field by the non-ferromagnetic debris dominated by the eddy current effect is far less than the enhancing of the magnetic field by the ferromagnetic debris dominated by the magnetization effect, so the inductive sensor can not identify the non-ferromagnetic debris with small size. This paper proposed a comprehensive method to improve the detection sensitivity of an inductive sensor for non-ferromagnetic debris. Based on the inductance and resistance parameters, the sensor’s detection limit for non-ferromagnetic debris has been enhanced. In addition, the magnetic field in the detection region is enhanced and focused by adding paramagnetic materials (a silicon steel strip was selected in this paper) in the coil inner hole so as to improve the detection accuracy. To further obtain better detection results, the frequency characteristics of the inductive sensor are studied, and the optimal frequency is selected.

## 2. Sensor Design and Detection Principle

### 2.1. Sensor Design

**Figure 1:**
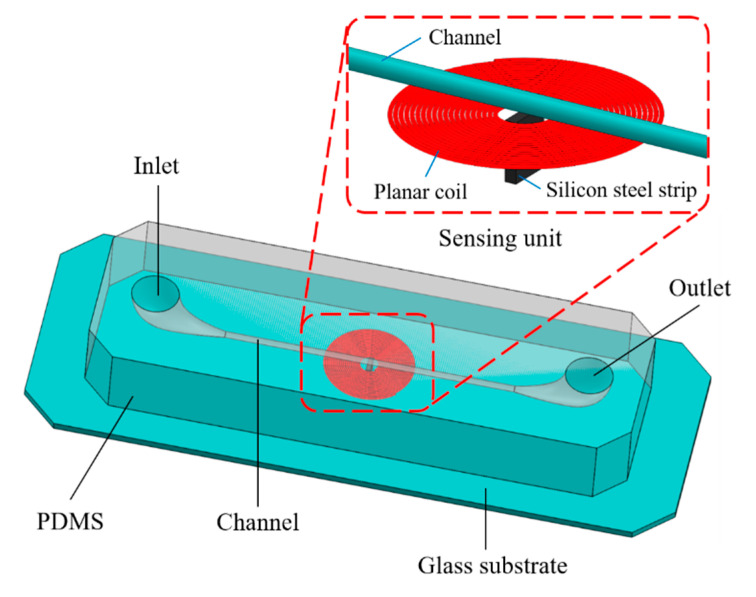
The structure of the inductive sensor.

The inductive debris sensor is shown in Figure 1. The designed sensor consists of a sensing unit, a detection channel, and a sensor substrate, and it is made using the mold-casting method. The sensing unit is composed of the planar coil inserted with a silicon steel strip in the inner hole, and the detection channel is close to the coil surface. This sensor structure could be used to enhance the magnetic field in the detection region and reduce the distance between the coil and the wear debris, so that the sensor is more sensitive. First, the copper wire (60 µm in diameter, with a thin insulation) is wound into a planar coil (4 layers, 20 turns per layer, inner diameter is 900 µm) by a winding machine (Shili SRDZ23-1B, Zhongshan ShiLi Wire Winder Equipment Co., Ltd., Zhongshan, China). Then, the channel inlet mold, channel mold (the 300 µm diameter steel wire), channel outlet mold, planar coil, and silicon steel strip (0.3 mm in thickness, 800 µm in width, 3 mm in length) were fixed using acrylic block and metallic glue, which form a basic mold. Next, polydimethylsiloxane (PDMS) was poured into the basic mold and cured in a heating oven (DZF-6020A, Bangxi Instrument Technology Co., Ltd., Shanghai, China). Lastly, the channel inlet mold, the channel mold, and the channel outlet mold were removed from the PDMS substrate. After the above steps, the fabrication of the sensor was completed.

### 2.2. Detection Principle

According to prior research [27], the impedance change caused by a non-ferromagnetic particle is

(1) $$Z\left(r_{p}\right)=\frac{U\left(r_{p}\right)}{I}=\frac{\oint_{c\mathrm{oil}}^{\;}-j\omega A_{p}\left(r\right)\cdot dl}{I}.$$

Here, $U\left(r_{p}\right)$ is the induced electromotive force of the coil, I is the current in the coil, ω is the angular frequency of the excitation alternating current, dl is a vector about the differential element of the wire in the direction of the conventional current, r is the arbitrary position vector of the coil, $r_{p}$ is the position vector of the particle center, $j^{2}=-1$. $A_{p}\left(r\right)$ is the magnetic vector potential distribution induced by the particle, which is given by

(2) $$A_{p}\left(r\right)=\frac{v\chi_{a}}{4\pi }B\left(r_{p}\right)\times \frac{r-r_{p}}{{\left|r-r_{p}\right|}^{3}}$$

where $B\left(r_{p}\right)$ is the magnetizing field and v is the volume of the non-ferromagnetic particle. $\chi_{a}$ is the magnetic susceptibility in an alternating magnetic field, which is given by

(3) $$\chi_{a}=\frac{3}{2}\frac{\left(2\mu_{r}+1-a^{2}k^{2}\right)\sin\left(ak\right)-ak\left(2\mu_{r}+1\right)\cos\left(ak\right)}{\left(a^{2}k^{2}+\mu_{r}-1\right)\sin\left(ak\right)-ak\left(\mu_{r}-1\right)\cos\left(ak\right)}\;.$$

here, $\mu_{r}$ is the relative permeability of the non-ferromagnetic particle, $\mu_{r}\approx 1$. a is the radius of particles, and *k* is given by

(4) $$k=\sqrt{-j\omega \mu_{r}\mu_{0}\sigma }\;$$

where σ is the conductivity of the non-ferromagnetic particle.

In the sensing unit, the magnetic field in the detection region is generated by the planar coil and silicon steel strip. Therefore, the magnetizing field $B\left(r_{p}\right)$ is

(5) $$B\left(r_{p}\right)=B_{C}\left(r_{p}\right)+B_{S}\left(r_{p}\right).$$

$B_{C}\left(r_{p}\right)$ is the magnetizing field generated by the planar coil, and $B_{S}\left(r_{p}\right)$ is the magnetizing field generated by the silicon steel strip.

The equivalent resistance change is the real component of impedance change.

(6) $$\Delta R=Re\left[Z\left(r_{p}\right)\right]$$

The equivalent inductance change is the imaginary component of impedance change.

(7) ΔL=Im[Z(rp)ω]

**Figure 2:**
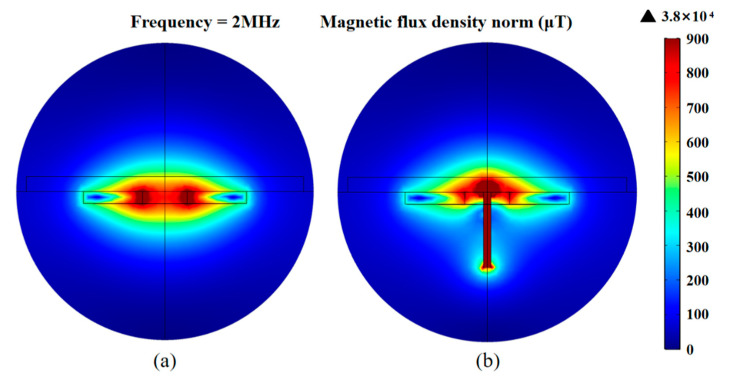
The simulation results of the magnetic field: (**a**) the magnetic field distribution of a planar coil; (**b**) the magnetic field distribution of a planar coil with a silicon steel strip.

In the alternating magnetic field, the eddy current effect in non-ferromagnetic particles reduces the magnetic field, and the metal particles change the skin effect and proximity effect of the coil. Therefore, non-ferromagnetic metal wear debris will generate negative inductive pulses and positive resistance pulses. As shown in Figure 2, the magnetic field change was simulated using COMSOL software. The average magnetic field intensity of a single planar coil in the detection region is 0.8 × 10^3^ μT, and the strongest magnetic field is distributed at the inner hole edge of the coil. After adding paramagnetic materials, the average magnetic field intensity in the detection region is 1.2 × 10^3^ μT, and the strongest magnetic field is located at the end of the silicon steel strip. This indicates that the addition of paramagnetic materials can enhance and aggregate the detection magnetic field.

## 3. Experiments and Discussions

### 3.1. Experimental Procedure

**Figure 3:**
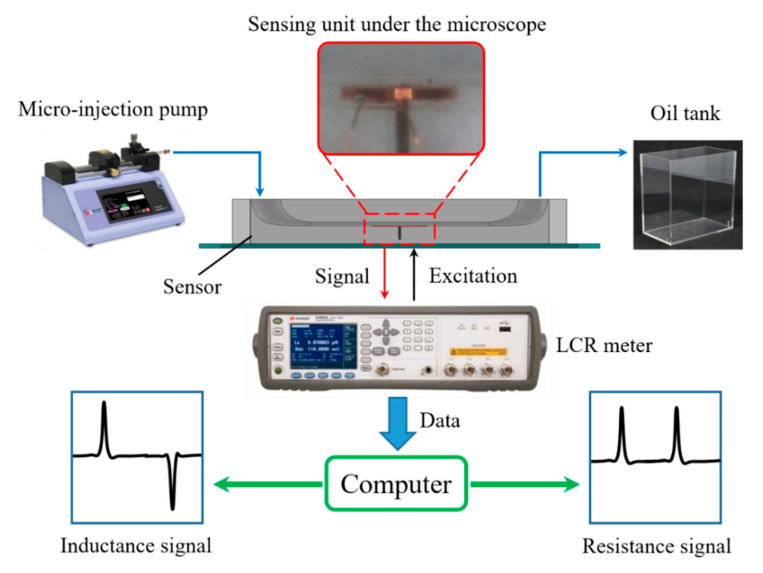
Experimental system.

The detection system is as shown in the Figure 3; it mainly includes a micro-injection pump (Harvard Apparatus B-85259, Harvard Apparatus, Holliston, MA, USA), an inductive sensor, a microscope (Nikon AZ100, Nikon, Tokyo, Japan), the an inductance (L), capacitance (C), and resistance (R) meter (Agilent E4980 A, Agilent Technologies Inc., Bayan Lepas, Malaysia), and a computer. 

The oil sample mixed with the spherical non-ferromagnetic metal particles is driven by the micro-injection pump to flow through the detection channel of the inductive sensor. The non-ferromagnetic metal particles that are measured by the microscope reciprocate through the inductive sensing unit by changing the injection direction of the micro-injection pump. Therefore, the same non-ferromagnetic metal particle can be detected multiple times and used in comparative experiments, so that the error caused by the shape characteristic of particles can be ignored. The inductance sensor is excited by the LCR meter with alternating current, and the LCR meter can collect the impedance data of the coil in real time and transmit it to the computer. The LabVIEW program and MATLAB program in the computer can process and analyze the data. In the experiment, the voltage of the LCR meter is set to 2.0 V, and the frequency is set to 0.2–2.0 MHz. The flow rate of the micro-injection pump is set to 300 μL/min.

### 3.2. Results and Discussion

**Figure 4:**
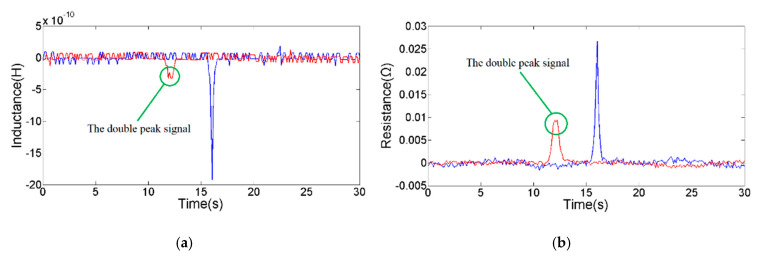
The comparison of experimental results; the blue line is the signal of the sensing unit, and the red line is the signal of the reference unit: (**a**) inductance detection signal of 124 µm copper particle; (**b**) resistance detection signal of 124 µm copper particle.

In the Detection Principle section, simulation studies show that the magnetic field strength and magnetic field distribution in the detection region are significantly enhanced and changed by the silicon steel strip. To explore the enhancement effect of the signal pulse amplitude, the coil without a silicon steel strip is selected as the reference unit to carry out the comparative experiment with the sensing unit designed in this paper. The 124 μm copper particles were selected under the microscope, and the particles were driven through the sensing unit and reference unit. As shown in Figure 4, the results of inductance detection and resistance detection are significantly improved after adding a silicon steel strip. The inductance and resistance signal noise are unchanged; the amplitude and SNR of the inductance signal are increased by 4.42 times; the amplitude and SNR of the resistance signal are increased by 1.89 times. In addition, when there is no silicon steel strip, a single particle will pass through the two strongest magnetic fields (the inner hole edge of the coil) and produce a double-peak signal. The double-peak signal is not conducive to analyzing and judging the particle size when multiple particles pass the detection region. The silicon steel strip in the coil focuses and enhances the magnetic field in the detection region, which prevents the appearance of a double-peak signal generated by a single particle.

**Figure 5:**
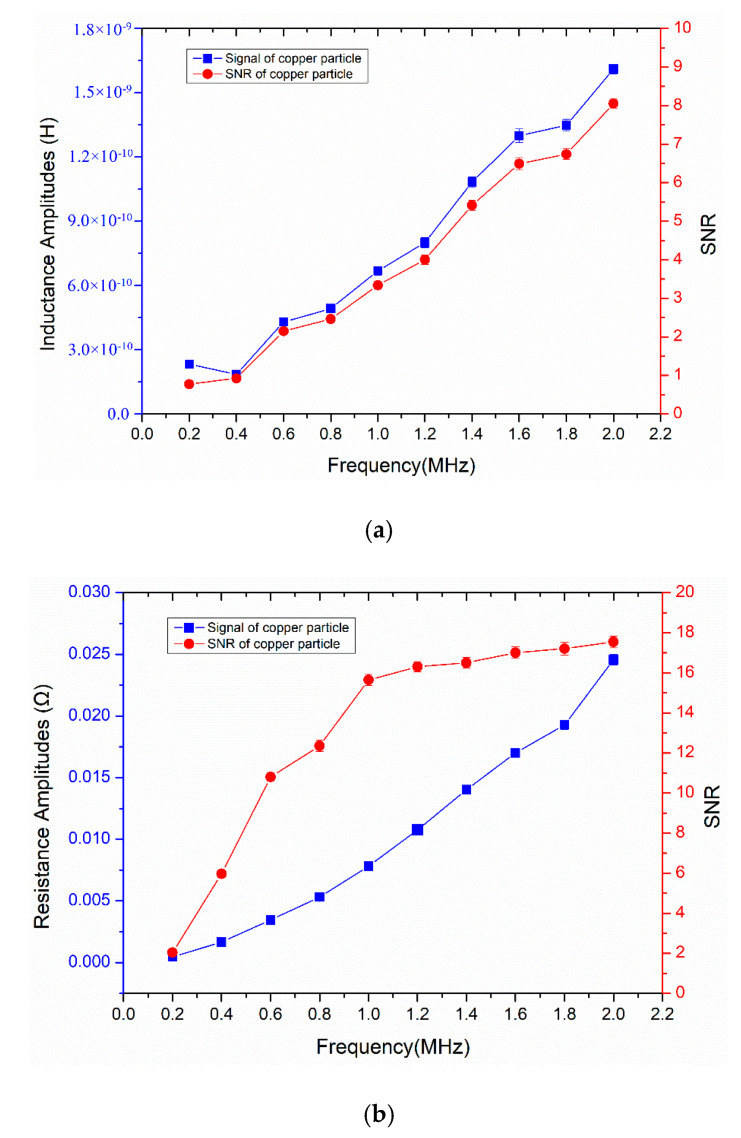
The characteristics of the detection result with the excitation frequency: (**a**) the average inductance amplitude and signal-to-noise ratio (SNR) of a 120 µm copper particle at different frequencies; (**b**) the average resistance amplitude and SNR of a 120 µm copper particle at different frequencies.

**Figure 6:**
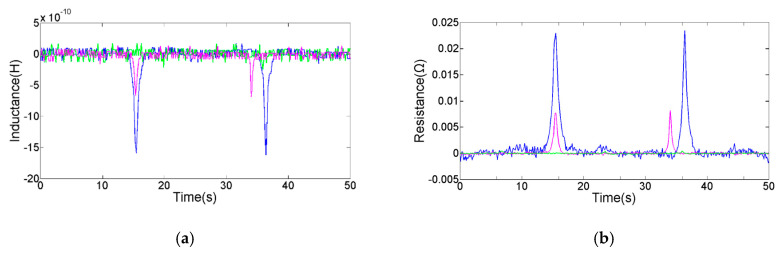
Comparison of detection results, the green line is the signal at 0.2 MHz, the red line is the signal at 1.0 MHz, and the blue line is the signal at 2.0 MHz: (**a**) the inductance signal of a 120 µm copper particle; (**b**) the resistance signal of a 120 µm copper particle.

Equation (1) shows that the detection results are affected by the excitation frequency. Therefore, we studied the characteristics of the inductance and resistance detection result with the excitation frequency. Under the microscope, a 120 µm copper particle was selected for the detection experiment; the frequency range of the LCR meter was set between 0.2 and 2.0 MHz (the step size is 0.2 MHz); and the copper particles were repeatedly passed through by controlling the direction of the micro-injection pump. As shown in Figure 5, the statistics of the experimental data are given. The amplitude and SNR of the inductance signal increase steadily with the frequency in the range of 0.4–2.0 MHz; although the inductance signal amplitude at 0.2 MHz is bigger than that at 0.4 MHz, the noise is higher (the inductance noise is 3.0 × 10^−10^ H at 0.2 MHz, the inductance noise is 2.0 × 10^−10^ H at other excitation frequencies); therefore, the inductance SNR increases with frequency. The resistance amplitude increases steadily with the frequency; the resistance SNR increases greatly in the range of 0.2–1.0 MHz; the increase of resistance SNR tends to be flat in the range of 1.0–2.0 MHz; the reason is that the increase rate of resistance noise is relatively slow at 0.2–1.0 MHz, but the rate is faster at 1.0–2.0 MHz (the skin effect and proximity effect of the coil will increase with excitation frequency, which change the resistance noise). As shown in the Figure 6, we compare the inductance and resistance signals obtained at 0.2, 1.0, and 2.0 MHz. At 2.0 MHz, it has the largest inductance and resistance signal amplitude (the average inductance amplitude is 1.61 × 10^−9^ H, the average resistance amplitude is 2.46 × 10^−2^ Ω), and the SNR is also the biggest. According to the analysis of the detection results, high-frequency alternating current excitation is beneficial to improve the SNR of inductance and resistance signals. The main reason is that in the alternating magnetic field, the eddy current effect generated inside the metal particles will increase with the frequency [28]. Therefore, the magnetic susceptibility of non-ferromagnetic particles increases with the frequency, and the change of coil impedance also increases. The selection of high-frequency excitation can further improve the detection ability of the inductive sensor for non-ferromagnetic metal debris.

**Figure 7:**
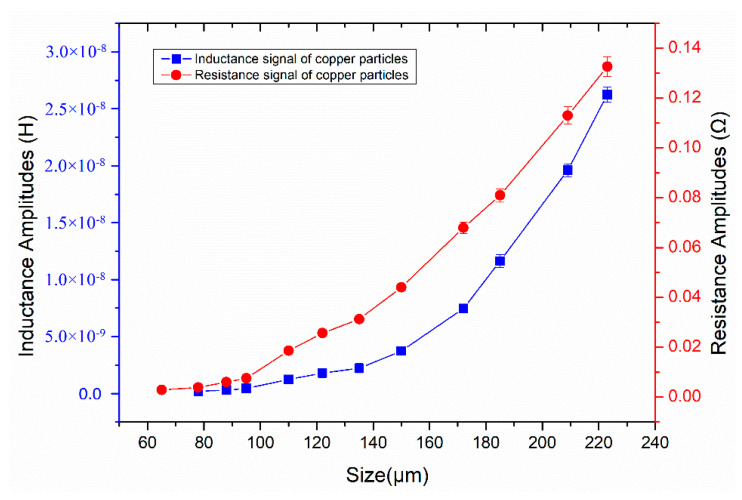
The relationship between average pulse amplitude and particle size.

**Figure 8:**
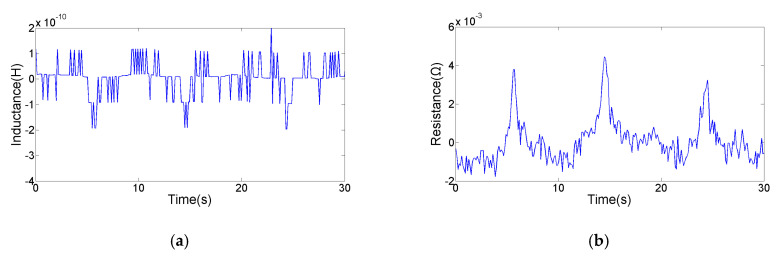
The detection result of 78 µm copper particle: (**a**) the inductance signal; (**b**) the resistance signal.

**Figure 9:**
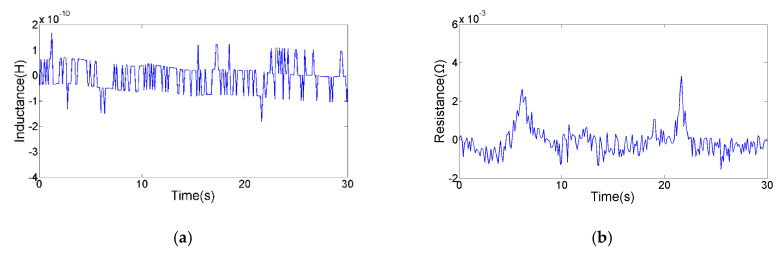
The detection result of a 65 µm copper particle: (**a**) the inductance signal; (**b**) the resistance signal.

The magnetic susceptibility and magnetic vector potential distribution induced by the particle are affected by the particle size. In order to obtain the corresponding relationship between the particle size and the average pulse amplitude, copper particles with different sizes were detected. As shown in the Figure 7, we calculated the average pulse amplitude generated by the different copper particles. Experimental results indicate that both inductance and resistance pulse amplitudes show a non-linear increase trend with particle size. Inductance and resistance pulses generated by debris with small size will be submerged in the signal noise, so that the SNR is too low to be identified. As shown in Figure 8, the smallest particle detected by the inductance parameter is 78 μm. The average inductance amplitude of the 78 μm copper particle is 2.0 × 10^−10^ H, while the average resistance pulse amplitude of the 78 μm copper particle is 3.88 × 10^−3^ Ω. As shown in Figure 9, the smallest particle detected by the resistance parameter is 65 μm. The inductance pulse of the 65 μm copper particle is submerged in inductance noise, while the average resistance amplitude of the 65 μm copper particle is 2.96 × 10^−3^ Ω. Compared with the inductance parameter, the resistance parameter can detect smaller non-ferromagnetic metal debris. Therefore, this impedance detection method involving inductance and resistance parameters is more conducive to improving the detection ability of the inductive sensor for non-ferromagnetic metal wear debris, so as to obtain more debris information. In addition, ferromagnetic and non-ferromagnetic wear debris can also be distinguished according to the characteristics of inductance and resistance signals [29].

## 4. Conclusions

The inductive debris sensor can effectively distinguish ferromagnetic and non-ferromagnetic metal particles, so it has wide application prospects in various fields. However, the insufficient detection ability of non-ferromagnetic debris restricts the comprehensive detection performance of the inductive sensor. Therefore, this paper proposes the methods of enhancing the magnetic field strength in the detection region, selecting the optimal excitation frequency, and using inductance and resistance parameters to improve the sensitivity and detection ability. Based on the theoretical research and experimental verification, the following conclusions are obtained.

(1) By adding paramagnetic materials in the coil inner hole to enhance the magnetic field strength of the detection region, the eddy current effect inside the non-ferromagnetic metal debris is more severe. Thereby, the detection sensitivity of the inductive debris sensor is significantly improved.

(2) The magnetic core makes the magnetic field distribution more concentrated, effectively preventing the generation of the double-peak pulse signal. It provides support for judging the particle sizes when multiple particles pass through the detection region at the same time.

(3) The eddy current effect generated inside the metal particles will increase with the excitation frequency. The inductive sensor that excited a high-frequency alternating current can not only obtain larger inductance and resistance amplitudes but also higher SNR.

(4) Compared with inductance parameter detection, resistance parameter detection has a better detection limit for non-ferromagnetic metal debris. This impedance detection method, which combines inductance and resistance parameters, can further improve the detection ability of the inductive debris sensor.
